# Age and hypertension strongly induce aortic stiffening in rats at basal and matched blood pressure levels

**DOI:** 10.14814/phy2.12805

**Published:** 2016-05-27

**Authors:** George Lindesay, Christophe Ragonnet, Stefano Chimenti, Nicole Villeneuve, Christine Vayssettes‐Courchay

**Affiliations:** ^1^ Cardiovascular Discovery Research UnitServier Research InstituteSuresnesFrance

**Keywords:** Age, aortic distensibility, aortic stiffness, echo tracking, hypertension, spontaneously hypertensive rat

## Abstract

Age and hypertension are major causes of large artery remodeling and stiffening, a cardiovascular risk factor for heart and kidney damage. The aged spontaneously hypertensive rat (SHR) model is recognized for human cardiovascular pathology, but discrepancies appeared in studies of arterial stiffness. We performed experiments using a robust analysis via echo tracking in 20‐week adult (*n* = 8) and 80‐week‐old SHR (*n* = 7), with age‐matched normotensive Wistar Kyoto rats (WKY,* n* = 6;6) at basal and matched levels of blood pressure (BP). After anesthesia with pentobarbital, abdominal aortic diameter and pressure were recorded and BP was decreased by clonidine i.v. At basal BP, aortic pulse distension, compliance, and distensibility (AD) were reduced and stiffness index increased with age and hypertension and further altered with age + hypertension. When BP was adjusted in SHR to that of normotensive rats (130 mmHg), there was no difference between 20‐week‐old SHR and WKY. Importantly, the age effect was maintained in both WKY and SHR and accentuated by hypertension in old rats. At 130 mmHg, with similar pulse pressure in the four groups, AD (kPa^−3^) = 24.2 ± 1 in 20 weeks WKY, 19.7 ± 1.4 in 20 weeks SHR, 12.4 ± 1.3 in 80 weeks WKY and 6.6 ± 0.6 in 80 weeks SHR; distension = 7.6 ± 0.4%, 6.7 ± 0.6%, 3.7 ± 0.3%, and 1.8 ± 0.2% in the same groups. In conclusion, reduced distensibility, that is, stiffening due to age is clearly shown here in both WKY and SHR as well as a synergistic effect of age and hypertension. This technique will allow new studies on the mechanisms responsible and drug intervention.

## Introduction

Increased large central artery stiffness is now recognized as a predictor of all‐cause mortality, fatal and nonfatal coronary event, and fatal strokes in not only disease populations such as essential hypertension (Laurent et al. 2001; Boutouyrie et al. 2002; Laurent et al. 2003), end‐stage renal disease (Blacher et al. 1998), or type II diabetes mellitus (Cruickshank et al. 2002) but also in the general population (Mitchell et al. 2010; Meaume et al. 2001). Therefore, the measurement of arterial stiffness through carotid‐femoral pulse wave velocity (PWV) is now recommended by the European society of Hypertension‐ European society of Cardiology Guidelines for the management of hypertension (Mancia et al. 2014) and reference values have been established (Reference Values for Arterial Stiffness' Collaboration, 2010; van Bortel et al. 2012). It is well established that both age and hypertension lead to increased arterial stiffness in man. Together, these factors behave synergistically to produce an even greater stiffening of compliant central arteries which lose their capacity to dampen the pulsatile forces of cardiac contraction. This stiffening arises due to a remodeling and disorganization of the vascular wall, including elastin fiber fracture (Lakatta et al. 1987), an increase in collagenous material, fibrotic components, calcium deposition (Laurent 2005), and alteration of the phenotype of vascular smooth muscle cells (Reusch et al. 1996; Lehoux and Tedgui 2003). Similar modifications are shown in rats, except less aortic calcification (Isabelle et al. 2012; Marque et al. 1999; Safar and Laurent 2003).

Despite the evidence derived from PWV measurements which show age and hypertension leading to arterial stiffening, discrepancies appear in studies of local stiffness in large arteries in human and animal models. These studies show that hypertension and aging may lead to either no change (Bézie et al. 1998; Laurent et al. 1994), increased (Labat et al. 2006), or decreased distensibility (Isabelle et al. 2012; Marque et al. 1999). These discrepancies likely arose due to differences in the techniques, arteries, ages, and blood pressure values. In humans, Paini et al. (2006) observed that the carotid artery does not accurately reflect aortic alterations in patients when aging is complicated with hypertension or diabetes. On the other hand, Stewart et al. (2006) showed that stiffness in man remained higher in hypertensives when their pressure was reduced to match the normotensives with sodium nitroprusside regardless of the artery analyzed. These data suggest that the artery studied and the way used to determine isobaric distensibility are determinant.

In rats, there is similar discord between findings made in studies which compare spontaneously hypertensive rats (SHR) and normotensive rats. In adult SHR, Labat et al. (2006) described an increased distensibility following a mathematical adjustment for BP, but van Gorp et al. (2000) described a reduced distensibility at low BP levels. Even taking into account the few existing experiments on aortic distensibility in old SHR, inconsistencies between the findings remain when isobaric distensibility is measured either via an echo tracking technique and the Langewouter model (Bezie et al. 1998; Labat et al. 2006) or using PWV (Marque et al. 1999; Isabelle et al. 2012). These data therefore do not lead to a conclusion concerning the effects of age and hypertension on aortic stiffness.

We previously evaluated aortic distensibility in a model of severe hypertension and aortic remodeling, the SHR‐LN, using the three methods, PWV (Isabelle et al. 2012), ultrasound echo tracking (NIUS) with the Langewouter model (Isabelle et al. 2012) and ultrasound echo tracking (ArtLab) with both systolo‐diastolic data and isobaric distensibility at matched mean BP (Vayssettes‐Courchay et al. 2011). The three methods indicated decreased distensibility in this model, but the later demonstrated a greater clarity and consistency between classic parameters of distensibility, compliance, stiffness as well as the distension wave analysis at basal and matched BP.

Therefore, it remained to quantify aortic stiffening in old hypertensive rats using this approach. Our objective was to assess whether this model is representative of the aortic remodeling seen in old, hypertensive humans.

## Methods

### Animals

This study was conducted in accordance with the European Community Guidelines for the use of experimental animals and was approved by the ethical committee on Animal Experiments of the Servier Research Institute. All animals were provided by Charles River (L'arbresle, France). Male Wistar Kyoto rats (WKY) and male spontaneously hypertensive rats (SHR) were age matched at 20 weeks (WKY *n* = 6; SHR *n* = 8) and 80 weeks (WKY *n* = 6, SHR *n* = 7). The animals had ad libitum access to both standard rat chow and water for the duration of their housing. The animals were housed together in their distinct groups with up to four animals per cage in a temperature‐controlled room (20–21°C) with a 12:12 h light:dark cycle.

### Hemodynamic measurements

**Figure 1:**
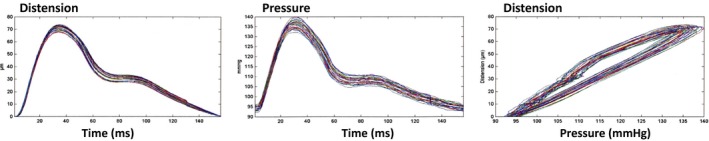
The figure shows a typical example of recordings over a period of 30 cardiac cycles for aortic distension, blood pressure, and the distension‐pressure loop (from an old WKY). These 30 beats are then average to represent 1 value for one rat.

Rats were anesthetized with an intraperitoneal injection of sodium pentobarbital (50 mg/kg: maintained with 5 mg/kg/h). The jugular vein was cannulated for constant administration of anesthesia and the penile vein was cannulated for administration of other drugs. The trachea was cannulated and ventilation was maintained with a pressure controlled respirator (Hallowell EMC, TEM) at a frequency of 60–70 cycles per minute and a pressure of 9–12 cmH_2_O. Body temperature was maintained at 38°C with a homeothermic blanket (Harvard) connected to a rectal probe. A microtip pressure catheter (Millar 1.2F) was inserted into the abdominal aorta via the right femoral artery. The blood pressure signal was visualized and analyzed with Biopac 4.2 Acknowledge acquisition and analysis system (CEROM). Aortic diameter was simultaneously measured with pressure using ultrasound. An ultrasound probe (L10‐5 40 mm 10 Mhz) was placed on the shaved skin on the side of the animal and was manipulated until a clear B‐mode image of the abdominal aorta and the intra‐arterial catheter was seen. A section of artery adjacent to the catheter was selected and subsequently analyzed in M‐mode. Vessel wall tracking technology (Art.Lab, Esaote, Netherlands) was used to measure the changes in arterial diameter for 6 sec (~30 cardiac cycles). The blood pressure signal was split and sent to a second computer containing Art.Lab to allow for blood pressure and diameter synchronization (Fig. 1). These data were subsequently analyzed using a specialized Matlab (Mathworks) program which integrates blood pressure and diameter data, and therefore allowed for arterial stiffness measurements. A more detailed description of both data acquisition via Art.Lab (Brands et al. 1999) and the specific analyses within Matlab (Vayssettes‐Courchay et al. 2011) have previously been published.

The parameters automatically calculated to determine the dynamic properties of the aortic wall are as follows: mean diameter and diastolic diameter (d*D*); mean distension (in *μ*m); compliance (Δ*A*/ΔP) in mm^2^/kPa, where *A* is the transsectional area of the vessel calculated from the diameter, and P is pressure; distensibility (Δ*A*/ΔP × *A*) in 1/kPa; and beta stiffness index {[d*D*ln(SAP/DAP)]/(s*D − *d*D*)},where s*D* is systolic diameter, SAP is systolic arterial pressure, and DAP is diastolic arterial pressure. Aortic distension was expressed in percent versus diastolic diameter (Δ*D *× 100/d*D*). The local pulse wave velocity (PWV) was calculated from √⌈(A/ΔP/*ρ*ΔA), *ρ *= blood viscosity.

In addition, from the beat‐to‐beat analysis, the pressure/time waveform, the distension/time waveform, and the hysteresis loop of distension pressure were constructed for each animal by averaging these data from 24 to 32 cardiac cycles and their respective area under the curve (AUC) were calculated. For the pressure wave and the distension (%) wave, the AUC values were adjusted for heart rate (AUC/msec = AUC × heart rate/60 × 10^3^) to avoid heart rate differences between groups (Vayssettes‐Courchay et al. 2011). The AUC of the distension pressure curve was calculated as the aortic wall viscosity (viscous energy) and as well as its percentage of the total area under the diastolic curve (energy stored during the distension phase + viscous energy) as previously described (Boutouyrie et al. 1997).

### Hemodynamic manipulations

In order to gain a greater understanding of the pressure‐dependent changes in arterial stiffness in the rat models used, blood pressure was changed using intravenous administration of clonidine (3 *μ*g/kg) which decreases blood pressure by inhibiting *α*_2_‐receptors in the brainstem, thereby decreasing peripheral sympathetic tone. A measurement was made at baseline blood pressure for each animal and again as blood pressure was slowly decreasing due to the action of clonidine. The discrete blood pressure chosen (after a measurement at baseline blood pressure) was: 130 mmHg for the SHR. The baseline blood pressure for an anesthetized WKY is approximately 130 mmHg, which allowed for a simple comparison with the SHR.

### Statistical analysis

All data were expressed as the mean ± the standard error of the mean (SE). The coefficient of variation measurement (%) was calculated. Then, each hemodynamic parameter was analyzed with a two‐way ANOVA of raw data followed by a Dunnett post hoc comparison on age and hypertension, first at basal blood pressure and again at matched blood pressures across all groups. The raw data residuals followed a normal distribution and the variances were homogeneous. Three parameters, *β*‐stiffness, distension wave AUC/msec, and % arterial wall viscosity did not follow a normal distribution without log10 transformation, which was followed by the two‐way ANOVA and Dunnett test. The differences were considered significant if *P* < 0.05 (SAS^®^ version .9.2).

Correlation analysis was performed for blood pressure, distensibility, stiffness index, and distension; Pearson r and p value were determined (GraphPad Prism software version 6.03).

## Results

### Hemodynamic effects at baseline blood pressure

Body weight in adult rats were similar (WKY: 340 ± 7 g and SHR: 334 ± 7 g). Old WKY had as expected a higher body weight (628 ± 14 g) than adult rats and old SHR (396 ± 7 g). Table 1 shows the hemodynamic parameters at baseline blood pressures across all groups. There was no difference in baseline blood pressure between the adult and old WKY while the adult SHR showed after anesthesia higher blood pressure than the old SHR. Despite these lower pressures, the old SHR showed higher aortic *β*‐stiffness index compared to the adult SHR. WKY showed an increase in aortic stiffness with age as determined by all measures of aortic stiffness. Similarly, hypertension at both ages induced an increased *β*‐stiffness index and decreased distensibility.

**Table 1:** Hemodynamic values and aortic properties

	WKY adult basal	SHR adult basal	WKY old basal	SHR old basal	SHR adult reduced BP	SHR old reduced BP
*N*	6	8	6	7	7	7
Mean arterial pressure (mmHg)	129 ± 2	203 ± 3#	128 ± 4	159 ± 7*, #	131 ± 1	132 ± 2
Systolic arterial pressure (mmHg)	156 ± 1	241 ± 5#	157 ± 5	187 ± 9*, #	161 ± 2	157 ± 3
Diastolic arterial pressure (mmHg)	109 ± 2	175 ± 2#	112 ± 4	138 ± 6*, #	110 ± 1	115 ± 2
Pulse pressure (mmHg)	47 ± 2	66 ± 4#	45 ± 2	49 ± 3*	51 ± 2	41 ± 2*
Heart rate (beats/min)	348 ± 16	407 ± 10#	408 ± 9*	366 ± 16*, #	339 ± 14	342 ± 13#
Diastolic diameter (*μ*m)	1344 ± 19	1307 ± 29	1691 ± 37*	1645 ± 37*	1206 ± 35#	1639 ± 17*
Distension (%)	7.6 ± 0.4	3.2 ± 0.2#	3.7 ± 0.3*	1.6 ± 0.1*, #	6.7 ± 0.6	1.8 ± 0.2*, #
Distensibility (1 × 10^−3^)	24.18 ± 0.86	7.51 ± 0.75#	12.41 ± 0.87*	4.97 ± 0.80*, #	19.66 ± 1.44#	6.57 ± 0.59*, #
Compliance (1 × 10^−3^)	37.35 ± 1.20	10.26 ± 0.77#	28.72 ± 1.94*	10.71 ± 2.99#	24.04 ± 1.49#	14.48 ± 1.43*, #
*β*‐Stiffness	5.00 ± 0.16	10.74 ± 0.88#	10.09 ± 0.71*	21.95 ± 1.73*, #	6.15 ± 0.42	19.98 ± 1.72*, #
Local pulse wave velocity	6.3 ± 0.1	11.6 ± 0.5	9.1 ± 0.4	14.6 ± 0.5	7.1 ± 0.2	12.8 ± 0.5 *, #
Distension wave AUC/msec	3.87 ± 0.22	1.6 ± 0.1#	1.61 ± 0.12*	0.8 ± 0.1*, #	3.24 ± 0.30	0.85 ± 0.11*, #
Pressure wave AUC/msec	20.3 ± 1.0	28.0 ± 1.8	16.3 ± 0.6	20.5 ± 1.5	20.8 ± 1.2	20.8 ± 1.0*
Arterial wall viscosity	58.39 ± 5.32	35.7 ± 2.9	29.14 ± 2.41*	12.5 ± 2.3*, #	54.33 ± 7.21	10.86 ± 2.41*, #
% Arterial wall viscosity	26.65 ± 0.80	27.7 ± 1.6	28.19 ± 0.85	25.1 ± 1.5	25.88 ± 0.54	23.17 ± 1.71#

Hemodynamic values are shown in the four groups at baseline, expressed as mean ± SE. *P < 0.05 for difference between age within strain, ^#^ *P* < 0.05 for differences between WKY and SHR at the same age *P* < 0.05. Reduced blood pressure after clonidine administration and isobaric parameters are indicated in right columns for adult SHR and old SHR.

Because of this difference in SHR groups, the first BP level analyzed in adult SHR after clonidine injection was 151 ± 3 mmHg. At this similar level of pressure, the old SHR have higher stiffness parameters and decreased distensibility parameters. The values for adult SHR at such blood pressure value were: *b*‐index = 6.7 ± 0.5, local PWV = 7.9 ± 0.3 m/sec) *D* = 15.6 ± 1.3 kPa*10^−3^, *C* = 19.4 ± 1.2 and distension = 5.7%.

### Hemodynamic effects at normotensive‐matched blood pressure

**Figure 2:**
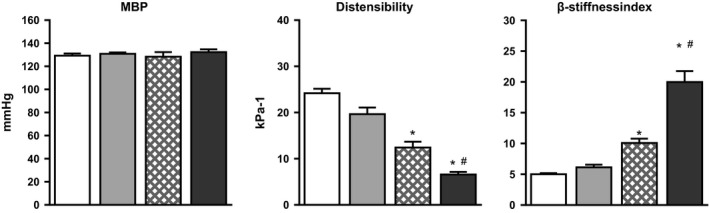
The figure shows that after clonidine administration (1.5 *μ*g/kg i.v.) in SHR, mean blood pressure (MBP) values are matched to those of WKY. Isobaric distensibility and *β*‐stiffness index in adult SHR (*n* = 7, gray bars) are no longer altered versus adult WKY (*n* = 6, white bars). However, both isobaric parameters remain altered in old WKY (*n* = 6, crossed bars) versus adult WKY and in old SHR (*n* = 7, black bars) compared to the other groups. Data are means ± SE. *P < 0.05 for difference between age within strain, ^#^ *P* < 0.05 for differences between WKY and SHR at the same age *P* < 0.05 (two‐way ANOVA of raw data followed by a Dunnett post hoc comparison on age and hypertension).

When blood pressure was reduced in both groups of SHR to match that of the baseline for normotensive rats (130 mmHg), we observed that the adult SHR no longer showed a difference in stiffness parameters (*β*‐stiffness index and local PWV) compared to the adult WKY; however, there was still a slight decrease in distensibility and compliance. The old SHR retained its previously observed increase in stiffness as compared with both the adult SHR and the old WKY with all parameters (Fig. 2).

There was a significant increase in diameter as a function of age with both the old WKY and old SHR. There was also a slight reduction in diastolic diameter in the adult SHR compared to the adult WKY. Finally, the old WKY had a higher heart rate than both the adult WKY and the old SHR (Table 1).

Correlations were calculated across all blood pressure values. MAP was correlated with distensibility, but not with *β*‐stiffness index (Pearson *r* = −0.50 and 0.10, respectively), however, *β*‐stiffness index and distensibility were correlated (Pearson *r* = −0.8). Distension was correlated with MAP (Pearson *r* = 0.34) across all groups except in old SHR.

### Analysis of pressure and distension waveforms

**Figure 3:**
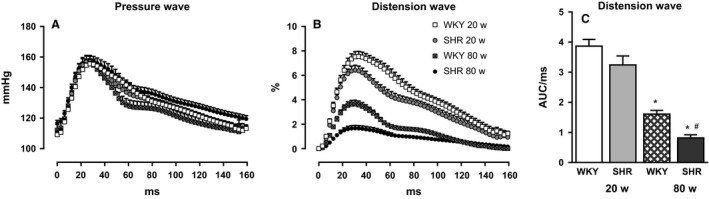
Comparison of pressure (A) and distension curves (B) at a MAP of 130 mmHg. The area under each curve of the distension wave, corrected for heart rate, is shown in part (C). With no difference in aortic pressure, both the old WKY (*n* = 6 crossed bars) and SHR (*n* = 7, black bars) display a lower total distension compared to the adult WKY (*n* = 6 white bars) and the old SHR displays a lower distension than the old WKY and adult SHR (*n* = 7, crossed bars). For clarity, only 1 out of 3 points are plotted. Data are means ± SE. *P < 0.05 for difference between age within strain, ^#^ *P* < 0.05 for differences between WKY and SHR at the same age *P* < 0.05.

**Figure 4:**
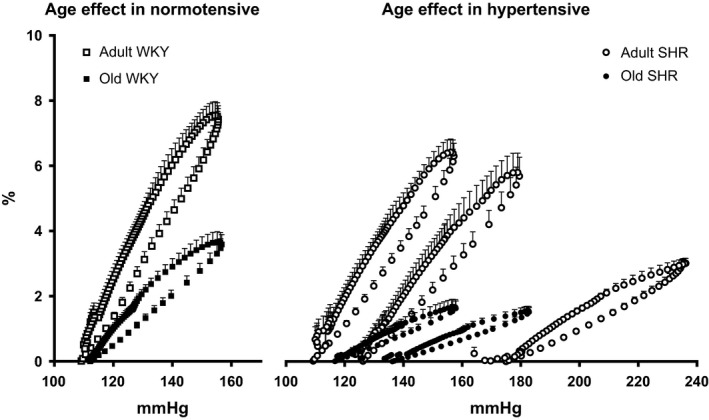
Pressure distension loops in adult and old normotensive and hypertensive rats. The adult SHR (*n* = 7, white circle) shows an increase in maximum distension as pressure is decreased while the old SHR (*n* = 7, black circle) is unchanged. The aged WKY (*n* = 6 black squares) loop shows a lowered distension despite similar mean and pulse pressure when compared to adult WKY (*n* = 6, white squares). The areas within the loops are shown in Table 1. Data are means ± SE. For clarity only 1 out of 3 points are plotted.

Figure 3 shows both the changes in aortic pressure and aortic distension throughout the cardiac cycle at a matched MAP of 130 mmHg. While there is no difference between the shapes of the pressure waveforms, there is a significant decrease in the magnitude of distension first in the old WKY with respect to the adult WKY and again a further decrease in total distension in the old SHR compared to both the adult SHR and the old WKY. The quantified area under the distension curve for these parameters reveals the same pattern. When expressed as pressure distension hysteresis loops (Fig. 4), one can see the differences in distensibility (Table 1) as represented by the slopes of each loop and arterial wall viscosity as the area of the loop. The AWV expressed as AUC within the loop was also strongly affected. While there was a strong correlation between the AWV and distensibility, when we corrected for the total area under the diastolic curve, the differences disappeared.

## Discussion

By measuring aortic stiffness at isobaric blood pressure, this study has quantified the contribution of age, hypertension, and age combined with chronic hypertension on aortic arterial stiffness. The stiffness measurements taken at basal blood pressure indicated both an effect due to different operational pressures and putative remodeling. Since these measurements were taken again at isobaric pressure, the changes remaining in arterial stiffness could only be accounted for by vascular remodeling. Specifically, we have found that aging leads to an increase in aortic stiffness and this stiffening is made worse when coupled with chronic hypertension in rats.

While the stiffening effects of aging and hypertension have been well described in humans as reported with aortic PWV (Reference Values for Arterial Stiffness' Collaboration, 2010), this effect does not hold true when assessing carotid (Laurent et al. 1994) or radial (Laurent et al. 1993) arterial stiffness with ultrasonographic devices. This suggests that either (1) these vascular beds are not ideal surrogates for the human aorta; or (2) the techniques used do not accurately assess aortic stiffness. Paini et al. (2006) found a strong correlation between regional aortic PWV (carotid‐femoral PWV) and local carotid PWV calculated from ultrasonic device in healthy patients, but this was diminished in essential hypertensives and patients with type 2 diabetes, indicating that these vessels change differently with disease. Using ultrasonic echo tracking on the carotid artery, Stewart et al. (2006) showed a decreased isobaric distensibility in hypertensive humans in contrast to (Laurent et al. 1994; Armentano et al. 1995) who did not observe changes in isobaric distensibility, each of these studies had a different technique to assess isobaric parameters. As Paini et al. (2006), Kawano et al. (2013) showed a reduced compliance in human when assessing aging alone. Therefore, it seems that both the artery studied and the way to evaluate isobaric parameters, which differ greatly in these studies (see below) may account for the discrepancies.

Similar differences are noted in rat models. A number of studies in spontaneously hypertensive rats (SHR) show that hypertension leads to a decrease (Zanchi et al. 1997; Stella et al. 1997; Labat et al. 2006), no change (Bézie et al. 1998; van Gorp et al. 1995; van Gorp et al. 2000), or an increase (Marque et al. 1999; Isabelle et al. 2012) in aortic stiffness.

In this study, the adult SHR had an increased *β*‐stiffness index and decreased compliance and distensibility at basal BP, but these changes were abolished or greatly reduced after BP reduction via clonidine administration. This is in agreement with the above studies which measured PWV (Marque et al. 1999; Isabelle et al. 2012) and confirm our previous results, obtained with another BP‐reducing agent, the calcium blocker diltiazem (Vayssettes‐Courchay et al. 2011). Despite the differences in the techniques used to assess stiffness, all the studies consistently show that in young adult SHR, there is a lack of pressure‐independent arterial stiffening. It has been suggested that the arterial wall thickening at this age, as in moderate hypertension in man is an active adaptation to delay stiffening (see (Laurent and Boutouyrie 2015).

This study shows that in aged SHR, stiffness and distensibility parameters are strongly altered when compared to both younger SHR and age‐matched normotensive rats at baseline blood pressure. Importantly, all these parameters remained altered following BP reduction in contrast to those measured in adult SHR. Together, these data separate the pressure‐dependent effects on arterial stiffness and also the stiffening due to remodeling of the vascular wall resulting from age and hypertension. This is in agreement with PWV measurements in aged SHR (Marque et al. 1999; Isabelle et al. 2012), but in contrast to the results from in SHR at 80 weeks old (Labat et al. 2006). We cannot comment on the differences between the later study (Labat et al. 2006) as the results for the pressure‐adjusted (done so via an unreported method) aortic stiffness were not shown.

In addition to hypertension‐induced stiffening with age, our present results showed that age, in the absence of hypertension was also able to produce a significant increase in arterial stiffness just as is seen in human aging (Reference Values for Arterial Stiffness' Collaboration, 2010; Kawano et al. 2013). Old normotensive populations represent an extremely important and rare cohort due to the positive feedback loop which exists between age‐induced vascular damage, aortic stiffening, and the development of hypertension (O'Rourke and Hashimoto 2007). Few studies have assessed the effects of advanced aging on aortic stiffness in normotensive rats, with again discrepancies in the results (Marque et al. 1999; Labat et al. 2006; Isabelle et al. 2012). Altogether, it appears that beyond the artery and the age of the groups studied, the manipulations performed to determine isobaric distensibility are crucial. Isobaric distensibility is determined either with a mathematical adjustment to BP, a pharmacologically induced decrease of BP to match those of normotensive rats or within the systolo‐diastolic changes of pressure with the Langewouter method. The later fits a pressure‐distensibility curve to the values of arterial pressure and diameter acquired throughout the cardiac cycle at a static mean arterial pressure (Laurent et al. 1994; Hayoz et al. 1992). Thereafter, points of overlapping pressure between groups are chosen to represent a common isobaric pressure. In hypertensive rats (Zanchi et al. 1997; Bézie et al. 1998) and humans (Laurent et al. 1994), the curves poorly overlap or do not overlap making isobaric comparison almost impossible to define. Safar in 1996 (Safar 1996) reviewed this technique and concluded that the curves generated between the systolic–diastolic range cannot be extrapolated; the only way to assess this is via vasoactive compounds within the same subject, a conclusion similar to ours (Vayssettes‐Courchay et al. 2011) with the stiffness assessment techniques used in this study.

This study used the centrally acting *α*_2_ agonist clonidine to reduced blood pressure via a decrease in sympathetic nerve activity (Schmitt et al. 1967). Unlike the vasodilatory effect seen following injection of a NO donor, sodium nitroprusside, (Glaser et al. 1995), clonidine did not cause an increase in aortic diameters at lower blood pressures. This implies that there was no vasomotor effect of clonidine on the aorta and removes the potential error in measuring a smooth muscle‐mediated change in aortic stiffness between our groups.

Our conclusions on age and hypertension stiffening are reinforced by the *β*‐stiffness index, whose equation takes into account BP and is described as less dependent on BP level (Hayashi et al. 2015), but was not evaluated in previous papers on aged SHR. Here, we have a strong correlation between the increases *β*‐stiffness index and decreased distensibility. The increase in *β*‐stiffness index in aged SHR is almost not reduced at all by acute BP reduction.

As previously (Vayssettes‐Courchay et al. 2011; Giannattasio et al. 2008), we report changes in aortic stiffness as represented by both the shape of the distension waveform and the distension pressure loop. Our results derived from the shape of the distension waveform at various blood pressures reinforce our conclusions about the effects of age and hypertension on arterial stiffness and are consistent with the findings on arterial stiffness in man (Stewart et al. 2006; Giannattasio et al. 2008). The distension waveforms alone, when shown alongside the matched blood pressures of the four groups also clearly reflect the effects of age and hypertension. This is important, since, assuming that one has equal blood pressure between groups, this measurement alone is sufficient to diagnose an artery as stiff.

From the distension pressure loop, it is possible to calculate the aortic wall viscosity by measuring the area within the hysteresis loops between systolic distension and diastolic distension. However, if one must correct for the total area under the diastolic component of the distension curve to determine the relative increase in energy dissipation (wall viscosity) as a function of total energy throughout the cardiac cycle as stated previously (Boutouyrie et al. 1997), no difference appears between viscosity in any of our groups probably due to the strong alteration of distensibility. In this study, it is not clear whether or not the hysteresis at systole is modified or not. In order to properly quantify this parameter, a more specific study should be designed which fully characterizes the influence of heart rate, pulse pressure dPdt, and distensibility on the hysteresis loop.

Ultimately, an assessment of the morphological changes of the arteries is essential for understanding the in vivo effects of aging and hypertension. Analysis of aortic morphology was not carried out during this study which focused on in vivo evaluations because our team and others previously conducted these assessments. When assessing the presence of aortic remodeling and fibrosis, we previously showed (Isabelle et al. 2012) that both old WKY and old SHR had higher intimal thickness and higher expression of transforming growth factor‐*β*, fibronectin, plasminogen activator inhibitor‐1 and connective tissue growth factor than their strain‐matched 20‐week‐old counterparts. All the aforementioned parameters were also increased in old SHR when compared with age‐matched normotensive WKY but not in 20‐week‐old SHR. Thus, even in slightly younger exemplars of the “old” WKY and SHR (55 weeks), the structural data were completely in agreement with the functional results obtained in this study. Moreover, we also showed that endothelial dysfunction, the other crucial component of arterial aging was significantly reduced in 55‐week‐old SHR and not in 20‐week‐old SHR Marque et al. in 1999 (Marque et al. 1999) showed that the reductions in elastin/collagen content across age were consistent between the two strains thus leading to their conclusion that this ratio alone is not the cause for the difference in stiffness with age, and instead hypothesized that it is due to a disorganization of the wall media. This disorganization may be initially mediated by changes in fibronectin levels in the young SHR which increases adhesion of the smooth muscle cells with the extracellular matrix ECM (Bezie et al. 1998) of which a large number of these connections occur between smooth muscles and the elastic lamellae (Bézie et al. 1998). It is conceivable that when these elastin layers are still intact at a young age, these connections lead to the mechanical adaption of the arterial wall thus accounting for the observed resistance to arterial stiffness seen in the adult SHR. However, with advancing age, and the eventual degradation of these elastic lamellae due to cyclic stress (Lakatta et al. 1987), this protection may no longer be provided and thus arterial stiffening occurs. An increase in fibronectin was observed in aged SHR (Bézie et al. 1998; Isabelle et al. 2012).

In conclusion, we have shown that aging leads to an increase in aortic stiffness in the rat and this stiffening is made worse when coupled with chronic hypertension. These results reflect the values typically obtained when assessing human aortic PWV under the same conditions. Therefore, the aged rat model may be considered as a viable surrogate for human aortic stiffness when assessing novel drugs to reduce arterial stiffness.

## Conflict of Interest

None declared.

## Supporting information




**Table S1.** Hemodynamic values and arotic properties expressed as mean and coefficient of variation.
