# Supplementary material for: Age and hypertension strongly induce aortic stiffening in rats at basal and matched blood pressure levels
Source: Physiol Rep. 2016 May 27;4(10):e12805. doi: 10.14814/phy2.12805 (PMC4886171; doi:10.14814/phy2.12805)
Supplement: Supplementary file 1 — Table S1. Hemodynamic values and arotic properties expressed as mean and coefficient of variation. [file PHY2-4-e12805-s001.docx]

**Supplemental data**

|  | **WKY adult**  **basal** | **SHR adult**  **basal** | **WKY old**  **basal** | **SHR old**  **basal** | **SHR adult**  **reduced BP** | **SHR old**  **reduced BP** |
| --- | --- | --- | --- | --- | --- | --- |
| N | 6 | 8 | 6 | 7 | 7 | 7 |
| Mean arterial pressure (mmHg) | 129± 3.45 | 202± 4.74 | 128± 7.53 | 159± 13.55 | 131± 2.58 | 132± 4.86 |
| Systolic Arterial Pressure (mmHg) | 155± 2.34 | 240± 5.79 | 157± 6.97 | 187± 13.57 | 161± 4.03 | 157± 5.30 |
| Diastolic Arterial Pressure (mmHg) | 108± 5.46 | 174± 3.95 | 111± 8.27 | 138± 13.19 | 110± 1.59 | 115± 4.62 |
| Pulse Pressure (mmHg) | 47± 9.62 | 66± 15.52 | 45± 10.29 | 49± 15.97 | 51± 13.67 | 42± 11.84 |
| Heart rate (beats/min) | 348± 10.99 | 407± 7.23 | 408± 5.23 | 366± 12.22 | 339± 12.06 | 342± 10.0 |
| Diastolic Diameter (µm) | 1343± 3.46 | 1307± 6.23 | 1691± 5.41 | 1645± 5.99 | 1206± 8.35# | 1639± 2.79 |
| Distension (%) | 7.6± 13.88 | 3.2± 15.66 | 3.7± 20.97 | 1.6± 23.06 | 6.7± 26.28 | 1.8± 34.60 |
| Distensibility (1x10^-3^) | 24.18± 9.72 | 7.51± 23.49 | 12.41± 25.31 | 4.97± 18.93 | 19.66± 20.70# | 6.57± 23.83 |
| Compliance (1x10^-3^) | 37.35± 7.84 | 10.26± 21.12 | 28.72± 16.51 | 10.71± 22.90 | 24.04± 17.59# | 14.48± 26.06 |
| β-Stiffness | 5.00± 0.16 | 10.74± 0.88 | 10.09± 17.34 | 21.95± 20.88 | 6.15± 19.31 | 19.98± 23.46 |
| Local pulse wave velocity | 6.3± 4.75 | 11.6±12.34 | 9.1± 11.97 | 14.6± 9.80 | 7.1± 10.01 | 12.8± 10.85 |
| Distension wave AUC/ms | 3.87± 14.21 | 1.55± 13.33 | 1.61± 18.53 | 0.8± 25.42 | 3.24± 25.95 | 0.85± 35.55 |
| Pressure wave AUC/ms | 20.3± 12.67 | 28.0± 17.92 | 16.3± 8.91 | 20.5± 19.24 | 20.8± 15.93 | 16.61± 15.28 |
| Arterial wall viscosity | 58.39± 22.30 | 35.7± 22.88 | 29.14± 20.21 | 12.5± 48.92 | 54.33± 37.54 | 10.86± 58.89 |
| % Arterial wall viscosity | 26.65± 7.34 | 27.7± 16.41 | 28.19± 7.34 | 25.1± 15.97 | 25.88± 5.93 | 23.17± 19.50# |

Hemodynamic values are shown in the four groups at baseline, expressed as mean±CV%. * p<0.05 for difference between age within strain, # p<0.05 for differences between WKY and SHR at the same age p<0.05. Reduced blood pressure after clonidine administration and isobaric parameters are indicated in right coloms for adult SHR and old SHR.
