# Supplementary material for: Age and hypertension strongly induce aortic stiffening in rats at basal and matched blood pressure levels
Source: Physiol Rep. 2016 May 27;4(10):e12805. doi: 10.14814/phy2.12805 (PMC4886171; doi:10.14814/phy2.12805)
Supplement: Supplementary file 2 [file PHY2-4-e12805-s002.docx]

Hemodynamic values and arotic properties expressed as mean and coefficient of variation.
